# Exotic stable cesium polynitrides at high pressure

**DOI:** 10.1038/srep16902

**Published:** 2015-11-19

**Authors:** Feng Peng, Yunxia Han, Hanyu Liu, Yansun Yao

**Affiliations:** 1 College of Physics and Electronic Information, Luoyang Normal University, Luoyang 471022, China; 2 Beijing Computational Science Research Center, Beijing 10084, China; 3 Geophysical Laboratory, Carnegie Institution of Washington, NW, Washington, D.C. 20015, USA; 4 Department of Physics and Engineering Physics, University of Saskatchewan, Saskatoon, Saskatchewan, S7N 5E2, Canada; 5 Canadian Light Source, Saskatoon, Saskatchewan, S7N 2V3 Canada

## Abstract

New polynitrides containing metastable forms of nitrogen are actively investigated as potential high-energy-density materials. Using a structure search method based on the CALYPSO methodology, we investigated the stable stoichiometries and structures of cesium polynitrides at high pressures. Along with the CsN_3_, we identified five new stoichiometric compounds (Cs_3_N, Cs_2_N, CsN, CsN_2_, and CsN_5_) with interesting structures that may be experimentally synthesizable at modest pressures (*i.e.*, less than 50 GPa). Nitrogen species in the predicted structures have various structural forms ranging from single atom (N) to highly endothermic molecules (N_2_, N_3_, N_4_, N_5_, N_6_) and chains (N_∞_). Polymeric chains of nitrogen were found in the high-pressure *C*2/*c* phase of CsN_2_. This structure contains a substantially high content of single N-N bonds that exceeds the previously known nitrogen chains in pure forms, and also exhibit metastability at ambient conditions. We also identified a very interesting CsN crystal that contains novel N_4_^4−^ anion. To our best knowledge, this is the first time a charged N_4_ species being reported. Results of the present study suggest that it is possible to obtain energetic polynitrogens in main-group nitrides under high pressure.

The pursuit for new and efficient energy source has always been a focus of scientific research. Nitrogen, which is abundant in nature, may be sought as a high-energy-density material (HEDM). This is possible if one can transform the diatomic N_2_ molecules into single- or double-bonded polynitrogen, utilizing the large energy difference between the single and double/triple bonds^1^,^2^,^3^,^4^,^5^,^6^,^7^,^8^,^9^. Solely single-bonded polynitrogen, for example, has an estimated energy capacity of 4.6 eV/mol, about three times that of the most powerful energetic materials known today^10^. The realization of polynitrogens has been actively experimented in nitrides, due to the fact that the charged nitrogen species in such materials often show improved kinetic stability over pure polynitrogens. Before 1999, the azide anion (N_3_^−^), which exists in metal azides (i.e., NaN_3_, Pb(N_3_)_2_), was the only known polynitrogen ion. Producing new polynitrogens involves substantial experimental difficulties due to the metastability of the products. However, in 1999, Christe *et al.* reported a successful synthesis of marginally stable N_5_^+^ AsF_6_^−^ crystal, which contains the second polynitrogen ion, the N_5_^+^ cation^11^. Subsequently, thermally more stable N_5_^+^ SbF_6_^−^ and N_5_^+^ Sb_2_F_11_^−^ have been synthesized^12^. Around the same time, the N_5_^−^ anion^13^, neutral N_4_ molecule^14^ have also been isolated experimentally. These successes led to an increasing interest in polynitrogen compounds and to the search of other stable polynitrongen forms in recent years.

Theoretical studies have often been employed to guide experiments and to identify unknown polynitrogens. In the past, many polynitrogen forms have been predicted to be metastable, from neutral (N_3_ to N_60_) to charged molecules (N_4_^2+^, N_5_^−^, N_6_^+^, N_6_^−^, N_6_^2+^); several of which have already been realized in experiments^15^,^16^,^17^,^18^. Prior to the synthesis of N_5_^+^ AsF_6_^−^, for example, quantum mechanical calculations^19^ already predicted that the N_5_^+^ could be detected experimentally. Previous theoretical studies of polynitrogens have primarily focused on single molecules, while the experimental realizations often proceeded in solid state, seeking for suitable matrices. Prediction of solid-state polynitrogen materials had been formidable due to the complexity of the potential-energy surface. Such a task only became possible recently attributing to newly developed heuristic algorithms and the high-performance computation capability. In recent studies^20^,^21^, a novel LiN_5_ crystal consisting of stable N_5_^−^ anions has been predicted, which represents one of the first predictions of the polynitrogen compounds beyond ordinary stoichiometries. The LiN_5_ crystal is energetically stable only at high pressures, but exhibit mechanical stability at ambient conditions which provides the possibility for its recovery. Moving forward, in the present study we investigated the closed-related Cs-N system. Since Cs has the lowest ionization potential in alkali metals, it could lose the valence electron more easily than Li. Electrons acquired by polynitrogen anions in the Cs-N crystals reduce the electrons sharing between nitrogen atoms which ultimately change the bonding order. Thus, one expects that mixing reactive Cs with nitrogen will result in more diverse structures and higher energy densities of the products.

The ambient-pressure chemistry for the Cs-N system has been well studied. The CsN_3_ is the only energetically stable phase, formed by the Cs^+^ cation and double-bonded N_3_^−^ anion^22^. Same N_3_^−^ anions also exist in other alkali metal azides (LiN_3_, NaN_3_, KN_3_). The N_3_^−^ has considerable metastability at ambient conditions which results in many applications for example as a propellant in automobile airbags. The decomposition of the N_3_^−^ in CsN_3_ is highly exothermic and also environmental friendly. Previous theoretical studies^23^ suggested that the energy density of CsN_3_ can be further enhanced at high pressure, where the nitrogen atoms form extended structures. Herein, we present a thorough theoretical investigation of the Cs-N system with other possible stoichiometries at ambient and high pressures, ranging from the Cs_3_N to the CsN_5_. By changing the density of the electron donors (Cs) in the system, one is able to manipulate the bond strength of the polynitrogen anions such that the single N−N bonds can be stabilized at much lower pressures than that required for pure nitrogen. Six novel stoichiometric Cs-N compounds with fascinating structures were predicted which may be synthesized at high pressure and recovered at ambient conditions. This study revealed the possibility of the formation of several new polynitrogen forms in solids, including tetrazadiene (N_4_), pentazole (N_5_), hexazine (N_6_), and extended chains (N_∞_). The bonding nature in these polynitrogens is of great important to nitrogen chemistry and to the understanding of metal-nitrogen interactions.

## Results and Discussion

**Figure 1:**
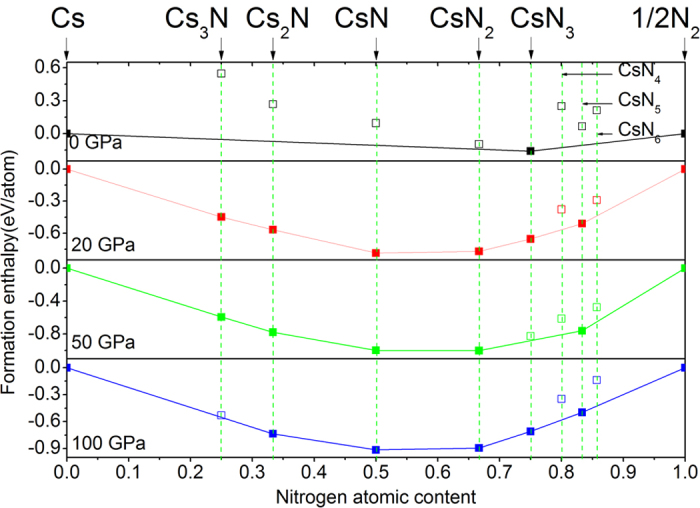
Relative enthalpies of formation of Cs-N phases with respect to elemental cesium and nitrogen solids. The convex hulls connecting stable phases (solid squares) are shown by solid lines. Unstable/metastable phases are shown by open squares.

**Figure 2:**
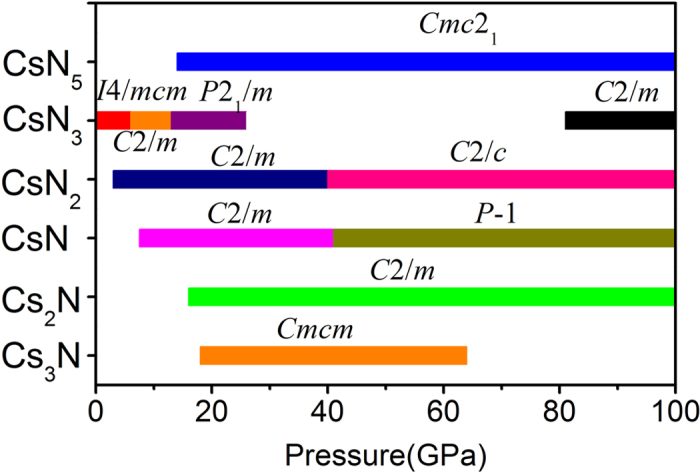
Predicted pressure-composition phase diagram of Cs-N crystal phases.

**Figure 3:**
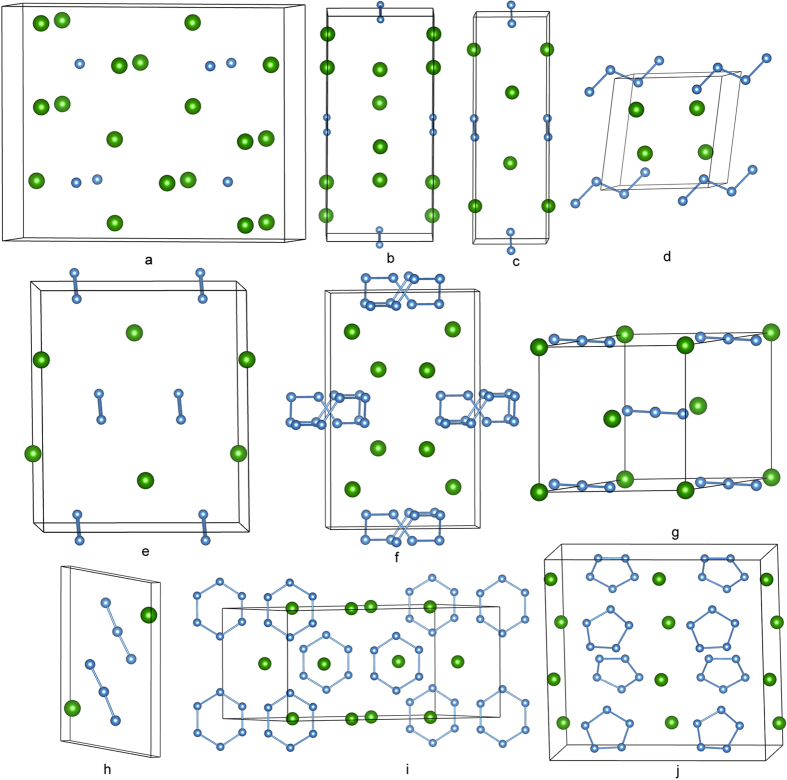
Structures of predicted stable Cs-N crystals. (**a**) *Cmcm* structure of Cs_3_N. (**b**) *C*2/*m* structure of Cs_2_N. (**c**) Low-pressure *C*2/*m* structure of CsN. (**d**) High-pressure *P*-1 structure of CsN. (**e**) Low-pressure *C*2/*m* structure of CsN_2_. (**f**) High-pressure *C*2/*c* structure of CsN_2_. (**g**,**h**) Low-pressure *C*2/*m* and *P*2_1_/*m* structures of CsN_3_. (**i**) High-pressure *C*2/*m* structure of CsN_3_. (**j**) *Cmc*2_1_ structure of CsN_5_. Nitrogen and cesium atoms are represented by blue and green spheres, respectively.

Figure 1 shows the enthalpies of formation, ∆*H*^f^, for the energetically most favorable Cs_1-*x*_N_*x*_ structures obtained in the structure search calculated at four pressures. The ∆*H*^f^ of each Cs-N structure was calculated relative to the enthalpy of cesium and nitrogen solids, using a fractional representation Cs_1-*x*_N_*x*_ (0 < *x* < 1), ∆*H*^f^(Cs_1-*x*_N_*x*_) = *H*(Cs_1-*x*_N_*x*_) − (1−*x*)*H*(Cs solid)—*xH*(N solid). The energetically most favorable structures known for solid cesium (bcc, fcc, *C*222_1_, *tI*4, *oC*16, and dhcp phases) and solid nitrogen (*α*-, *γ*-, *ε*-, and cubic gauche phases) were used as reference structures in their corresponding stable pressure ranges. In Fig. 1, the energetically stable phases at each pressure are shown by solid squares, which are connected by the convex hull (solid lines), whereas the unstable or metastable phases are shown by open squares. At ambient pressure, the only stable stoichiometry revealed in the structure search is the experimentally known CsN_3_^24^. At high pressures, five new stoichiometries, Cs_3_N, Cs_2_N, CsN, CsN_2_, and CsN_5_, previously unknown for the Cs-N system, were predicted to become energetically stable in different pressure ranges. Detailed pressure-composition phase diagram for the Cs-N system is presented in Fig. 2, noting here the predicted structure changes in CsN_3_, CsN_2_, and CsN at high pressure. Specifically, the predicted pressure ranges of stability are, 18 to 64 GPa for Cs_3_N, 16 to 100 GPa and above for Cs_2_N, 7.5 to 100 GPa and above for CsN, 4 to 100 GPa and above for CsN_2_, 0 to 26 GPa, and 81 to 100 GPa and above for CsN_3_, and 14 to 100 GPa and above for CsN_5_. It may strike as a surprise that energetically new stoichiometries, other than the commonly known CsN_3_ are calculated to be stable at high pressure. On the face of it, one sees the enigma of these stoichiometries violating the ‘octet rule’ for main-group elements. However, a closer look into the crystal structures reveals that all predicted stoichiometries are suited well with different bonding patterns of the polynitrogens, and can be explained by traditional electron count. The calculated phonon dispersion relations also confirmed the mechanical and dynamical stability of these structures (Supplementary Figs 1–7). The crystal structures of the predicted Cs-N phases are presented in Fig. 3a–j, whereas the crystallographic parameters are provided in Supplementary Table 1. In what follow these structures will be analyzed, and their relevance to energy storage applications will be discussed.

### Cs-N phases analogous to known metal nitrides

**Figure 4:**
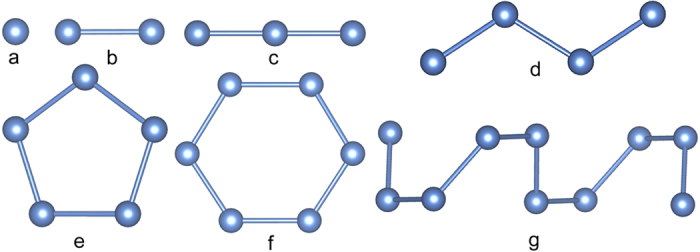
Forms of polynitrogen discovered in the Cs-N crystals. (**a**) Isolated N^3–^ in the Cs_3_N. (**b**) Diazene N_2_^2−^ and hydrazine N_2_^4−^ in CsN and Cs_2_N. (**c**) Azide N_3_^−^ in CsN_3_. (**d**) Tetrazadiene N_4_^4−^ in CsN. (**e**) Pentazole N_5_^−^ in CsN_5_. (**f**) Hexazine N_6_^2−^ in CsN_3_. (**g**) Spiral nitrogen chains in CsN_2_.

In the Cs-rich end, the Cs_3_N crystal adopts an orthorhombic *Cmcm* structure (Fig. 3a) between 18 and 64 GPa. Chemical structure of Cs_3_N is very similar to that of lithium nitride Li_3_N^22^. In the Cs_3_N, nitrogen atoms form N^3−^ anions with fully occupied subshells. The N-N separations in the *Cmcm* structure are large, *i.e.*, 3.16 Å at 20 GPa, which limits its capability of forming polynitrogens. It does, however, have an extremely strong nitrogen base, which, if can be recovered at ambient conditions, may be sufficient to deprotonate hydrogen leading to possible applications. The CsN crystal adopts a monoclinic *C*2/*m* structure between 7.5 and 44 GPa (Fig. 3c) in which the nitrogen atoms form double-bonded N_2_^2−^ anion (Fig. 4b). The N-N bondlength in the *C*2/*m* structure is 1.21 Å (at 20 GPa), which is a typical value for a double bond. The calculated Mayer bond order (MBO)^25^ for the N_2_^2−^ anion in the CsN is 2.20, also consistent with double bonding (Table 1). Same N_2_^2−^ anion has been known to exist in alkaline earth diazenides^26^,^27^,^28^,^29^. A related N_2_^4−^ anion, or deprotonated hydrazine (N_2_H_4_), was found in the *C*2/*m* structure of Cs_2_N (Fig. 3b). The *C*2/*m* structure of Cs_2_N is structurally similar to the *C*2/*m* phase of CsN, with one additional Cs atom at the 4i Wyckoff position. The two nitrogen atoms in the N_2_^4−^ are nearly single-bonded, as shown by the MBO value of 1.34. Calculated N-N bond length in Cs_2_N is 1.34 Å (at 20 GPa), similar to the value in the cubic gauche phase of nitrogen. The N_2_^4−^ have been synthesized in noble metal pernitrides^30^,^31^,^32^; many of the products exhibit remarkable properties (e.g., superconductivity, low compressibility) and, therefore, are often considered as technologically important materials.

**Figure 5:**
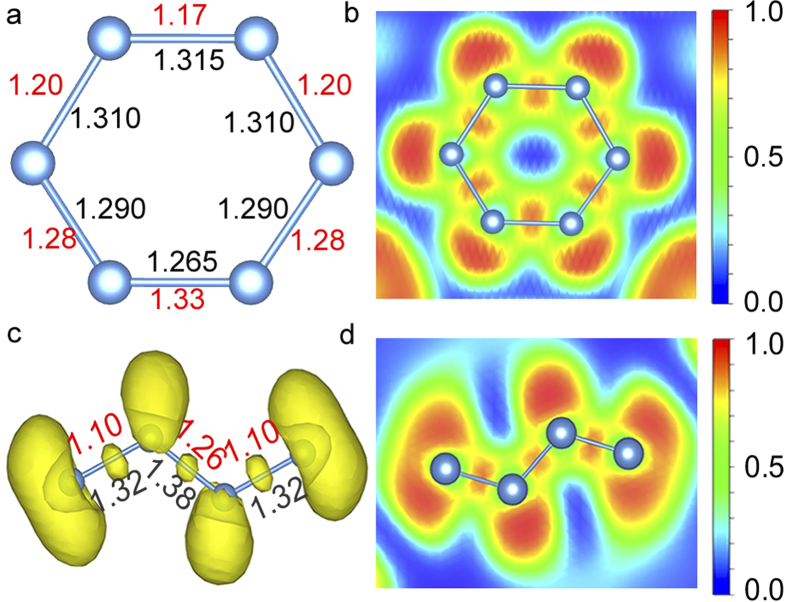
(**a**) The planer N_6_^2−^ anion isolated from the *C*2/*m* structure of CsN_3_ at 100 GPa. (**b**) The ELF map shown the (1 1 –2) cross-section of the *C*2/*m* structure of CsN_3_ at 100 GPa. (**c**) The ELF isosurface (value = 0.82) illustrated on a plane containing N_4_^4−^ in the *P*-1 structure of CsN at 50 GPa. (**d**) The ELF map shown on the (2 –2 –1) cross-section of the *P*-1 structure of CsN at 50 GPa. Numbers next to the N-N bond represent the bondlength (black, in Å) and MBO value (red), respectively.

At ambient pressure, the CsN_3_ crystal has the *I*4/*mcm* structure with azide N_3_^−^ anion (Fig. 4c). The double-bond feature of the N_3_^−^ is shown nicely by the calculated MBO value of 2.10 (Table 1). At high pressure, the *I*4/*mcm* structure is predicted to transform to a *C*2/*m* structure (Fig. 3g) at 7 GPa and then to a *P*2_1_/*m* structure (Fig. 3h) at 16 GPa (Fig. 2). These results are in good agreement with previous studies^23^,^33^. Another *C*2/*m* structure is predicted to have lower enthalpy than the *P*2_1_/*m* structure above 49 GPa (Fig. 3i). Energetically, however, the CsN_3_ becomes less stable already at 26 GPa with respect to the decomposition of CsN_2_ and CsN_5_, but regains the stability at above 81 GPa. Interestingly, if we treat the N_6_ as a unity located at its center of mass, the *C*2/*m* structure of CsN_3_ and the *C*2/*m* phase of Cs_2_N would have the same nitrogen sublattice and Cs positions. In the *C*2/*m* structure, the nitrogen atoms form cyclic N_6_^2−^ anions^34^,^35^,^36^,^37^. A neutral cyclic N_6_ molecule is the nitrogen analogue of benzene which has a planar D_6_ _h_ symmetry (Fig. 4f). In the CsN_3_, the charged N_6_ has a planar distortion for non-aromatic π system which results in four non-equivalent N-N bonds (Fig. 5a). The bonding pattern of the N_6_^2−^ is illustrated by the electron localization function (ELF) map^38^ drawn on the plane (Fig. 5b). The regions with the maximum ELF values on the plane are identified as six σ-bonds, and six lone pairs. Above and below the plane, the π electrons have cyclic delocalization counterbalanced by the positively charged nitrogen cores. Such arrangement of charges induces an electric quadrupole along the 6-fold axis of the N_6_^2−^, which may interact favorably with the Cs^+^ located on the axis. Similar cation-π interactions should also present in the high-pressure phases of LiN_3_ and KN_3_^34^,^36^,^37^. The calculated MBO values for the N-N bonds in the N_6_^2−^ anion are between 1.17 and 1.33 (Fig. 5a), slightly stronger than single bonds (1.0).

**Figure 6:**
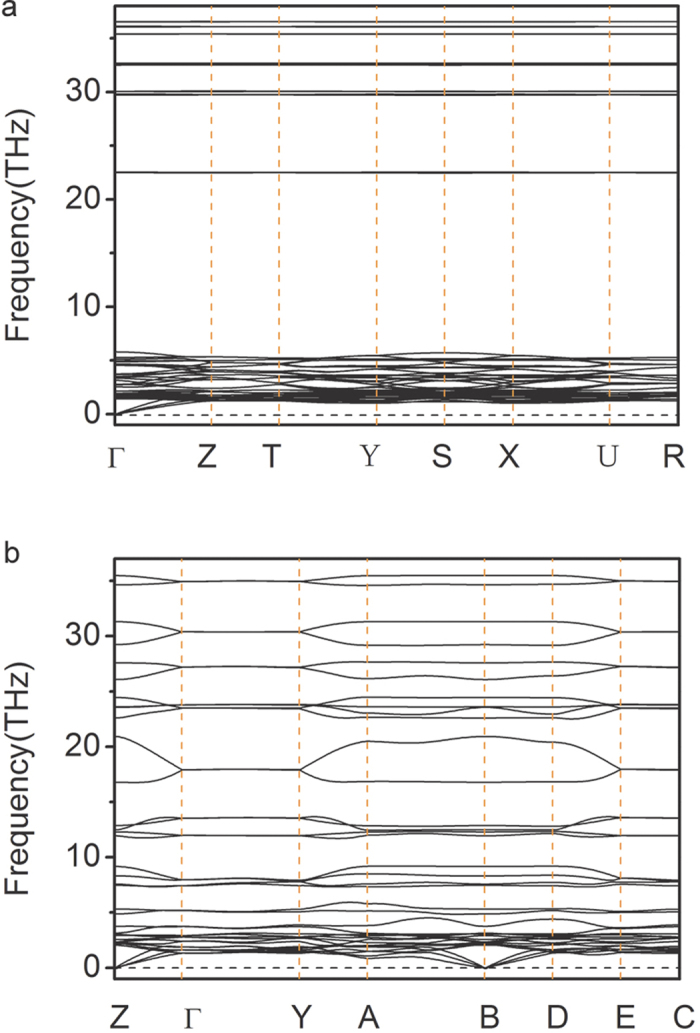
Phonon dispersion curves for the *Cmc*2_1_ structure of CsN_5_ (**a**) and *C*2/*c* structure of CsN_2_ (**b**) and at ambient pressure.

The CsN_5_ crystal forms a *Cmc*2_1_ structure (Fig. 3j) that is stable above 14 GPa and metastable at ambient pressure (Fig. 1), as the fact that phonon calculations carried out at ambient pressure in Fig. 6a reveal no imaginary modes. Its chemical structure is similar to that of the LiN_5_ we previously studied^20^. The nitrogen atoms in CsN_5_ form a planar N_5_^−^ anion of the D_5_ _h_ symmetry (Fig. 4e). Within the plane the N atoms are bonded by five σ-bonds. Above and below the plane the six π electrons form an aromatic set of π bonds that stabilizes the ring. Calculated N−N distances in the N_5_^−^ are of the same length, *i.e.*, 1.33 Å at ambient pressure, which is an intermediate between the single (1.45 Å) and double bonds (1.20 Å). Calculated MBO value of 1.43 also confirms this bonding nature (Table 1).

### CsN phase with energetic N_4_^4**−**^ anions

At pressures higher than 44 GPa, we identified a very interesting CsN crystal (Fig. 3d) that contains novel N_4_^4−^ anion (Fig. 4d). To our best knowledge, this is the first time a charged N_4_ species being reported in literature. The neutral N_4_ molecule has been successfully isolated in the gas phase a decade ago^14^. Dissociation of the N_4_ molecule was found to be highly exothermic, releasing ~ 800 kJ energy per mole. Such high energy content, if realized in a controlled manner, would place N_4_ among modern high-energy materials such as TATB, RDX, and HMX^39^. On the other hand, the lifetime of the N_4_ molecule is only around 1 microsecond, which limits its practical usages. Compared with the neutral counterpart, the charged N_4_^4−^ as predicted in the CsN crystal is substantially stabilized by strong cation-anion interactions, which is expected to have an extended lifetime. Furthermore, due to the additional electrons acquired from Cs, the N_4_^4−^ anion has a lower degree of electrons-sharing compared with the neutral counterpart, and therefore has a higher energy content.

The predicted N_4_^4−^ anion in CsN has an open-chain structure (Fig. 4d). Its Lewis structure has two terminal single-bonds (σ) and one internal double-bond (σ and π). The average bonding strength of the N_4_^4−^ is weaker than that of the neutral N_4_^40^. The bonding pattern of the N_4_^4−^ is illustrated by the ELF isosurface and cross section (Fig. 5c,d). The maximum of the ELF in N_4_^4−^ appears as lobes outside the nitrogen atoms, which are identified as the lone pairs. Between the nitrogen atoms the smaller high ELF regions correspond to three σ bonds. The π electrons are delocalized and distributed among nitrogen atoms, which is not directly visualized by the ELF but can be inferred from the similar bond lengths and strengths of the three N-N bonds (Fig. 5c). Clearly, all three N-N bonds in the N_4_^4−^ (1.10, 1.10, and 1.26) are slightly stronger than the single bonds (1.0) but much weaker than the double bonds (2.0). As well, the N-N bond in the middle of the molecule (1.26) appears to be slightly stronger than the two terminal counterparts (1.10), showing some resemblance to the Lewis structure. In bulk, crystalline CsN is a semiconductor with a very small, indirect band gap of 0.25 eV (Supporting Information).

### CsN_2_ phase with linear-polymeric nitrogen

**Figure 7:**
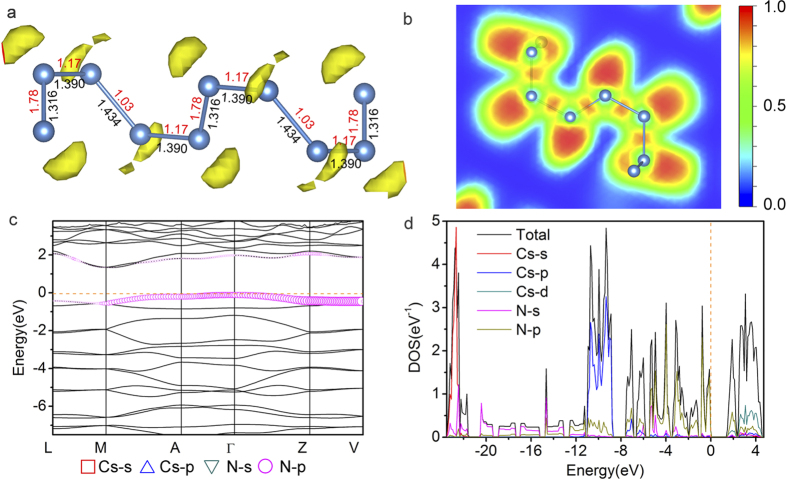
(**a**) The ELF isosurface (value = 0.93) illustrated on a plane containing N_8_^4−^ in the *C*2/*c* structure of CsN_2_ at ambient pressure. Numbers next to the N-N bond represent the bondlength (black, in Å) and MBO value (red), respectively. (**b**) The ELF map shown on the (1 1 –1) cross-section of the *C*2/*c* structure of CsN_2_ at ambient pressure. (**c**,**d**) Electronic band structure and projected DOS of the *C*2/*c* structure calculated at ambient pressure.

The CsN_2_ crystal is predicted to become energetically stable near 4 GPa. Between 4 and 40 GPa, CsN_2_ adopts the *C*2/*m* structure in which the nitrogen atoms still keep the diatomic form (Fig. 3e). Above 40 GPa, CsN_2_ transforms to the *C*2/*c* structure (Fig. 3f). In the *C*2/*c* structure the nitrogen atoms form one-dimensional helical chains that extend infinitely in the crystal, which is the only polymeric form of nitrogen discovered in the present study (Fig. 4g). One-dimensional chains of similar geometry have been predicted to exist in pure oxygen under high pressure^5^. According to the Zintl-Klemm rule, the negatively charged nitrogen can behave like oxygen under certain conditions. The repeating unit of the chain in the *C*2/*c* structure contains eight nitrogen atoms (Fig. 7a). In its Lewis structure, an extending N_8_^4−^ unit should have 6 single bonds and 2 double bonds, forming a 3:1 ratio. This assignment is confirmed by the calculated MBO values for the four independent N-N bonds in the chain, e.g., 1.03, 1.17, 1.17 and 1.78, respectively (Fig. 7a). The calculated bond distances are 1.43, 1.39, 1.39, and 1.32 Å, respectively, also correspond with a 3:1 ratio, although the differences are less distinct than those in the bond orders. The predicted *C*2/*c* phase of CsN_2_ is an insulator, as illustrated by the calculated band structure and electronic density-of-states (DOS) (Fig. 7c,d). The calculated band gap is indirect, with the magnitude of ~1.3 eV. The valence states occupied by nitrogen are separated from each other and grouped into subsets, which, in the order of increasing energy, are characteristic of 2s*σ*_g_/2s*σ*_u_*, 2p*σ*_g_/2p*π*_u_/2p*π*_g_* states. Notable contributions of nitrogen to the bands above and below the band gap are clearly seen in Fig. 7c.

Significantly, phonon calculations carried out at ambient pressure in Fig. 6b reveal no imaginary modes for the *C*2/*c* phase of the CsN_2_, suggesting that it is both mechanically and dynamically stable and might be quench recoverable. Furthermore, the minimum pressure required for the synthesis of this phase, ~40 GPa, is well within the current capability of high-pressure techniques. Nitrogen chains with such high content of single N-N bonds are not only promising for energy storage applications, but also of great impact in the rational design of polynitrogens. Polymeric nitrogen phases that are consisting of one-dimensional chains have been extensively studied for decades^2^,^3^. For pure nitrogen, however, electron count requires all such chains to have alternating single and double bonds which fixes the ratio of single bonds to double bonds to 1:1. The present study suggests that one may be able to increase the content of the single bonds simply by mixing the chains with electropositive metals. The negatively charged nitrogen chains are also stabilized by strong cation-anion interactions^41^ and therefore are expected to be more stable than their neutral counterparts.

## Methods

The search for energetically stable crystalline structures was made on various stoichiometry of CsN_x_ (*x* = 1/3, 1/2, 1, 2, 3, 4, 5 and 6) using simulation cells containing up to four formula units. Structure searches for all stoichiometries were carried out at four pressures, e.g., 0, 20, 50, and 100 GPa, using the particle swarm optimization methodology as implemented in the CALYPSO code^42^,^43^. In recent studies, it was shown that the approach was successful on the prediction of high pressure structures on both elemental and binary compounds, such as dense oxygen^44^, Bi_2_Te_3_^45^, and Xe-Fe complex^46^. Total energy calculations, geometrical optimizations, and electronic structure calculations were performed within the framework of density functional theory (DFT) using the VASP (Vienna Ab Initio simulation package) program^47^. Exchange and correlation of the electrons were treated by the generalized gradient approximation with the Perdew-Burke-Ernzerhof functional^48^. The projector-augmented wave (PAW) method^49^ was employed with the Cs and N potentials adopted from the VASP potential library. The Cs and N potentials have 5s^2^5p^6^6s^1^ and 2s^2^2p^3^ as valence states, respectively, and use an energy cutoff of 600 eV for the planewaves. A dense *k*-point grid^50^ with the spacing of 2π × 0.03 Å^−1^ was used to sample the Brillouin zone which was shown to yield excellent convergence for total energies (within 1 meV/atom).

## Conclusion

New compounds containing polymeric forms of nitrogen (polynitrogens) have been actively investigated as potential high-energy-density materials over the past decades. The energy contents of the polynitrogens result from the large differences in bond dissociation energies between single, double and triple N-N bonds. This fact makes the polynitrogen compounds metastable and highly endothermic. In the present study, swarm-intelligence structure searches were carried out to predict stable stoichiometries and structures of cesium polynitrides at high pressures. The goal is to discover metastable Cs_1-*x*_N_*x*_ structures containing polynitrogens that are energetically stable at high pressures. Along with the known CsN_3_, we found five more stoichiometries, namely, Cs_3_N, Cs_2_N, CsN, CsN_2_ and CsN_5_, to also become stable provided suitable pressure conditions. In the predicted crystals, the Cs atoms behave as electron donors whose concentration strongly influences the N-N bonding. This enables the nitrogen atoms to adopt versatile forms in molecules and extended structures, ranging from small clusters made of a few atoms (N_2_, N_3_, N_4_, N_5_, N_6_) to one-dimensional infinite chains (N_∞_). In particular, the N_4_^4−^ anion and the N_∞_ chain as found in the high-pressure phases of CsN and CsN_2_ contain an exceptionally high content of the single N-N bonds, which, if realized in controlled manner, may find applications as high energy carriers. At pressures above 40 GPa, the CsN_2_ structure with the N_∞_ chains is energetically stable, suggesting that it may be prepared by high-pressure synthesis. Moreover, this structure is mechanically stable at ambient conditions which may make an ambient-pressure recovery possible. The present study provides new insights to the understanding of polynitrogens and encourages experimental exploration of these promising materials in the future.

## Additional Information

**How to cite this article**: Peng, F. *et al.* Exotic stable cesium polynitrides at high pressure. *Sci. Rep.* **5**, 16902; doi: 10.1038/srep16902 (2015).

## Supplementary Material

Supplementary Information

## Figures and Tables

**Table 1:** Calculated Bader charges (captured electrons per N atom) and Mayer bond orders for the N–N bonds in predicted Cs-N phases at various pressures.

Phase	Nitrogen form	Charges (e)	Bond order
Cs_2_N (*C*2/*m*)@50 GPa	N_2_	0.96	1.34
CsN (*C*2/*m*) @20 GPa	N_2_	0.64	2.20
CsN (*P*-1) @50 GPa	N_4_	0.66	1.26, 1.10
CsN_2_ (*C*2/*c*)@0 GPa	Chains	0.39	1.03, 1.17, 1.78
CsN_3_ (*I4/mcm*)@0 GPa	N_3_	0.29	2.10
CsN_3_ (*C*2/*m*)@100 GPa	N_6_	0.26	1.17–1.33
CsN_5_ (*Pmc*2_1_)@0 GPa	N_5_	0.17	1.37–1.46
