# Supplementary material for: Exotic stable cesium polynitrides at high pressure
Source: Sci Rep. 2015 Nov 19;5:16902. doi: 10.1038/srep16902 (PMC4652274; doi:10.1038/srep16902)
Supplement: Supplementary Information [file srep16902-s1.pdf]

Supplementary information

Exotic stable cesium polynitrides at high pressures

Feng Peng[1,2], Yunxia Han[1], Hanyu Liu[3], Yansun Yao*[4,5]

[1]College of Physics and Electronic Information, Luoyang Normal University, Luoyang 471022, China

[2]Beijing Computational Science Research Center, Beijing 10084, China

[3]Geophysical Laboratory, Carnegie Institution of Washington, NW, Washington, D.C. 20015, USA

[4]Department of Physics and Engineering Physics, University of Saskatchewan, Saskatoon, Saskatchewan, S7N 5E2, Canada

[5]Canadian Light Source, Saskatoon, Saskatchewan, S7N 2V3 Canada

*Corresponding author E-mail: yansun.yao@usask.ca

Supplementary Figures

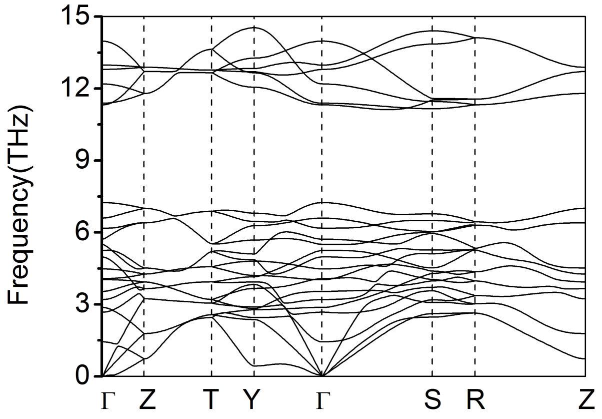

Fig. S1. Phonon dispersion curves of Cs_3N in the *Cmcm* phase at 50 GPa. The absence of any imaginary frequency in the whole Brillouin zone demonstrated that *Cmcm* structure of Cs_3N is dynamically stable.

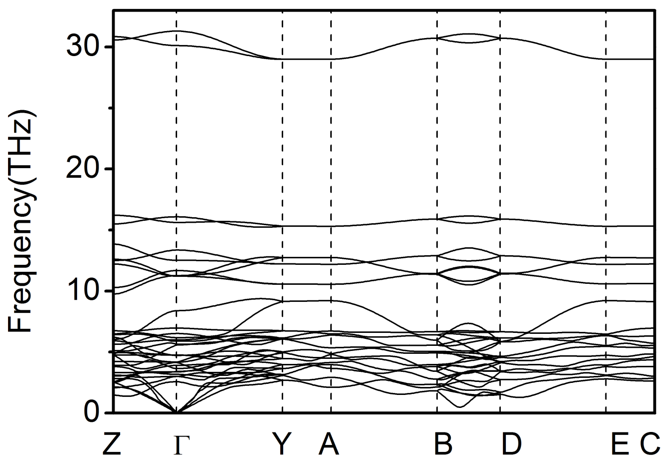

Fig. S2. Phonon dispersion curves of Cs_2N in the *C*2/*m* phase at 50 GPa. The absence of any imaginary frequency in the whole Brillouin zone demonstrated that *C*2/*m* structure of Cs_2N is dynamically stable.

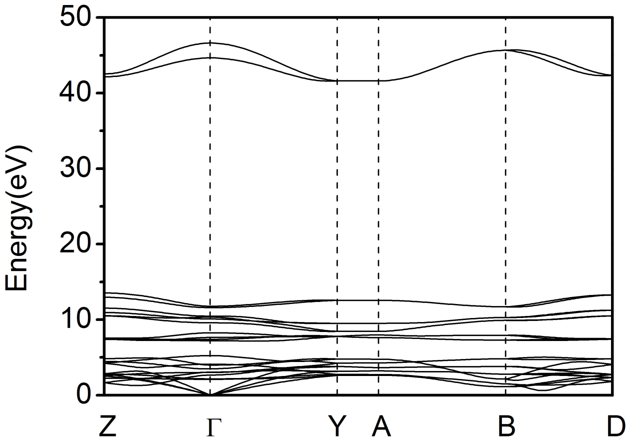

Fig. S3. Phonon dispersion curves of CsN in the *C*2/*m* phase at 20 GPa. The absence of any imaginary frequency in the whole Brillouin zone demonstrated that *C*2/*m* structure of CsN is dynamically stable.

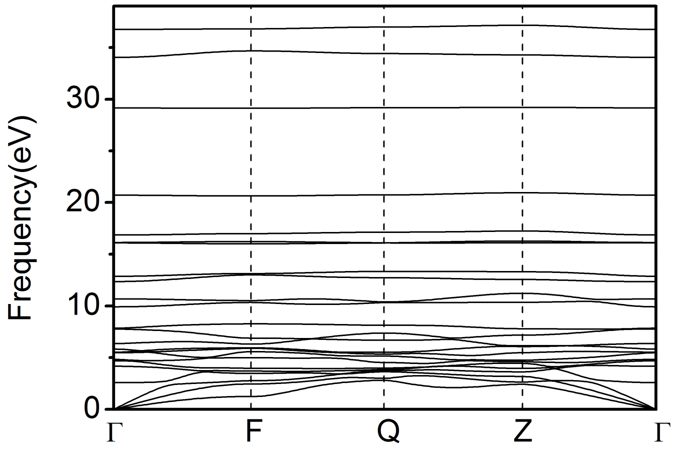

Fig. S4. Phonon dispersion curves of CsN in the *P*-1 phase at 50 GPa. The absence of any imaginary frequency in the whole Brillouin zone demonstrated that *P*-1 structure of CsN is dynamically stable.

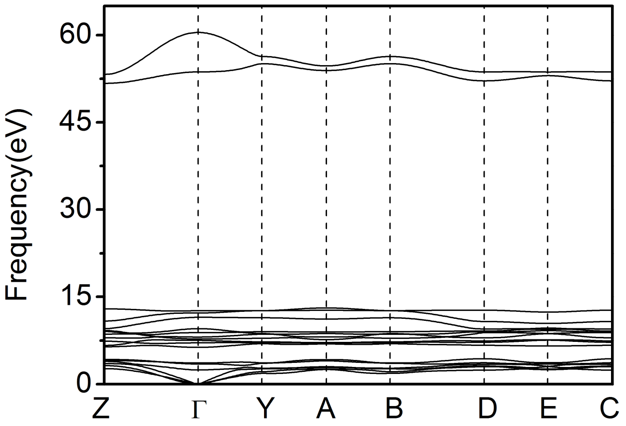

Fig. S5. Phonon dispersion curves of CsN_2 in the *C*2/*m* phase at 20 GPa. The absence of any imaginary frequency in the whole Brillouin zone demonstrated that *C*2/*m* structure of CsN_2 is dynamically stable.

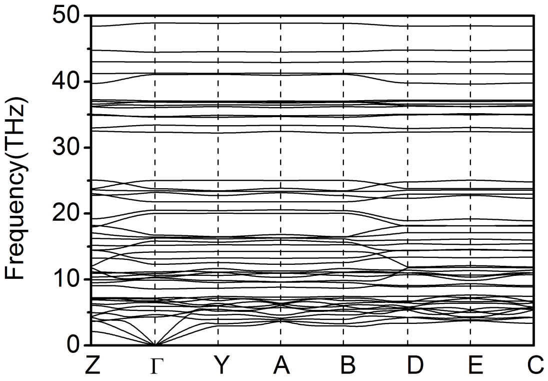

Fig. S6. Phonon dispersion curves of CsN_3 in the *C*2/*m* phase at 100 GPa. The absence of any imaginary frequency in the whole Brillouin zone demonstrated that *C*2/*m* structure of CsN_3 is dynamically stable.

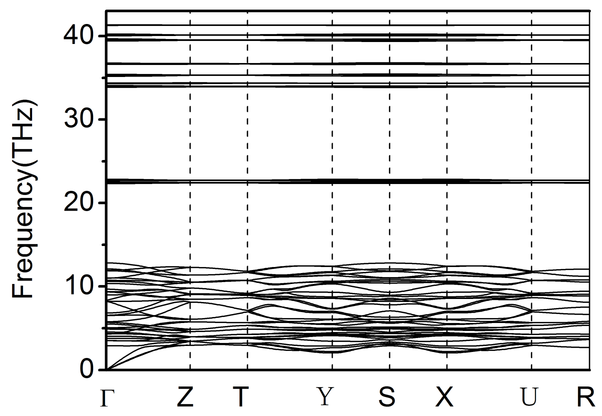

Fig. S7. Phonon dispersion curves of CsN_5 in the *Cmc*2_1 phase at 50 GPa. The absence of any imaginary frequency in the whole Brillouin zone demonstrated that *Cmc*2_1 structure of CsN_5 is dynamically stable.

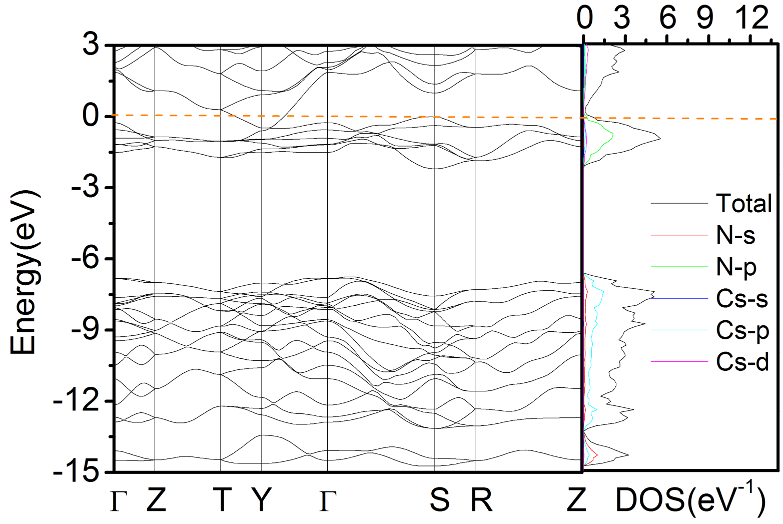

Fig. S8. Electronic band structure (left panel) and projected density of states (PDOS, right panel) of Cs_3N with *Cmcm* symmetry at 50 GPa. The dashed line indicates the Fermi energy.

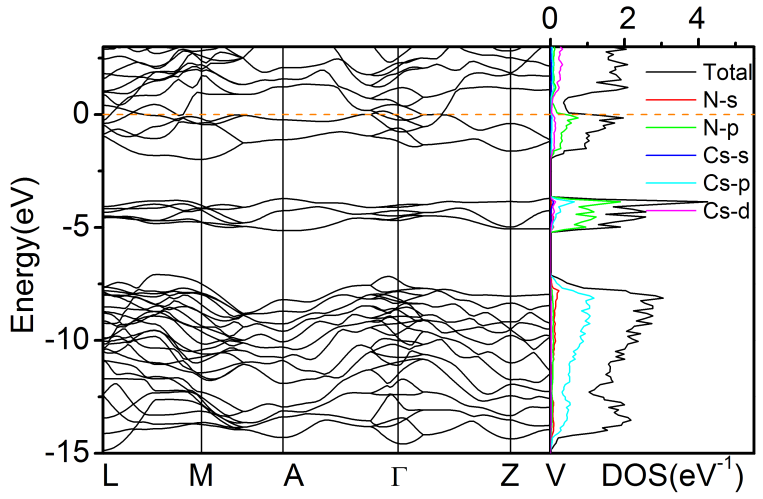

Fig. S9. Electronic band structure (left panel) and projected density of states (PDOS, right panel) of Cs_2N with *C*2/*m* symmetry at 50 GPa. The dashed line indicates the Fermi energy.

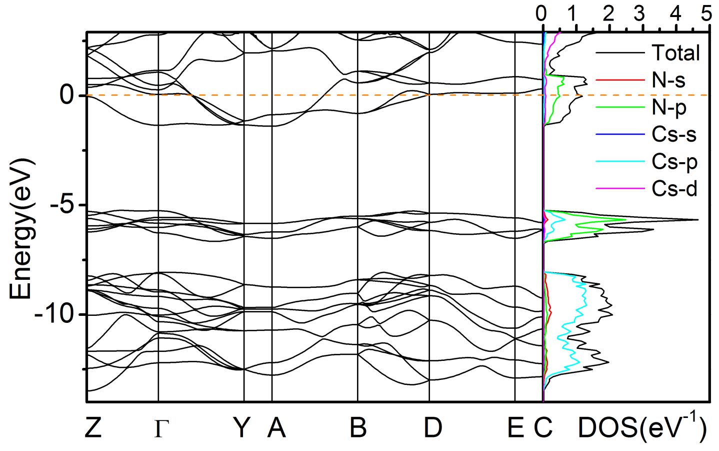

Fig. S10. Electronic band structure (left panel) and projected density of states (PDOS, right panel) of CsN with *C*2/*m* symmetry at 20 GPa. The dashed line indicates the Fermi energy.

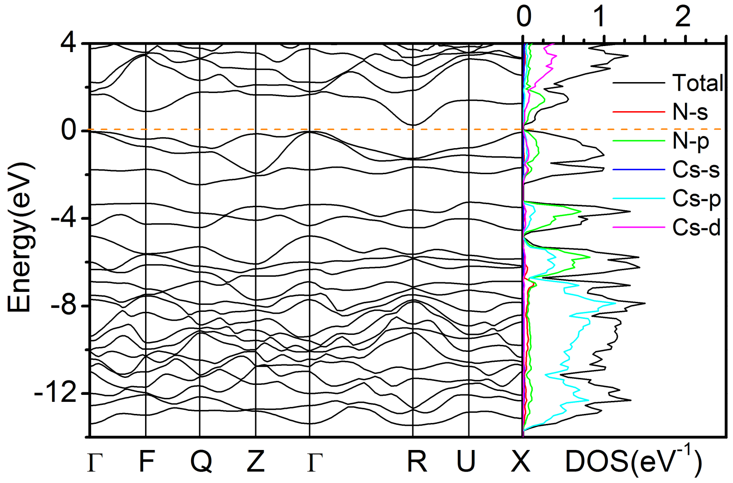

Fig. S11. Electronic band structure (left panel) and projected density of states (PDOS, right panel) of CsN with *P*-1 symmetry at 50 GPa. The dashed line indicates the Fermi energy.

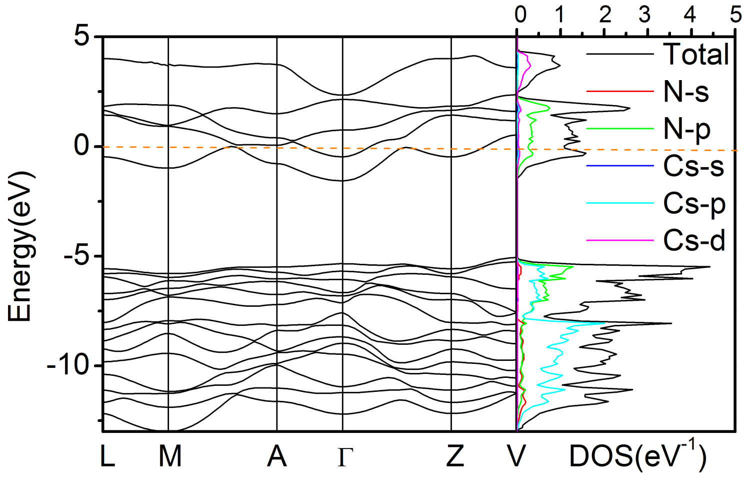

Fig. S12. Electronic band structure (left panel) and projected density of states (PDOS, right panel) of CsN_2 with *C*2/*m* symmetry at 20 GPa. The dashed line indicates the Fermi energy.

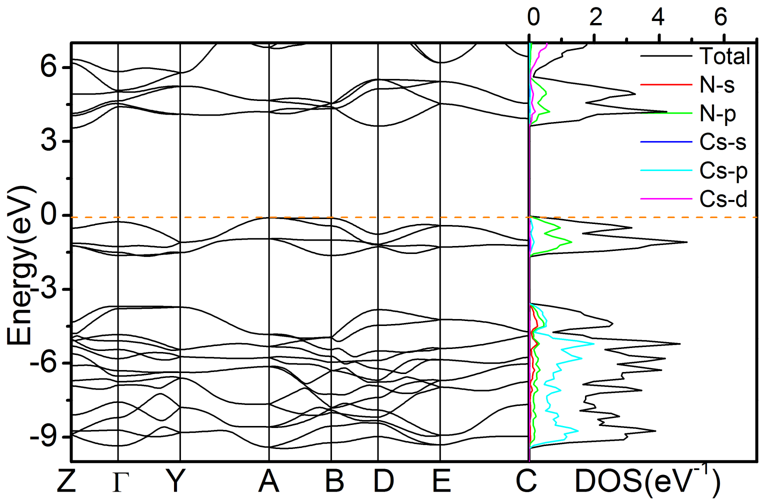

Fig. S13. Electronic band structure (left panel) and projected density of states (PDOS, right panel) of CsN_3 with $P2_1/m$ symmetry at 20 GPa. The dashed line indicates the Fermi energy.

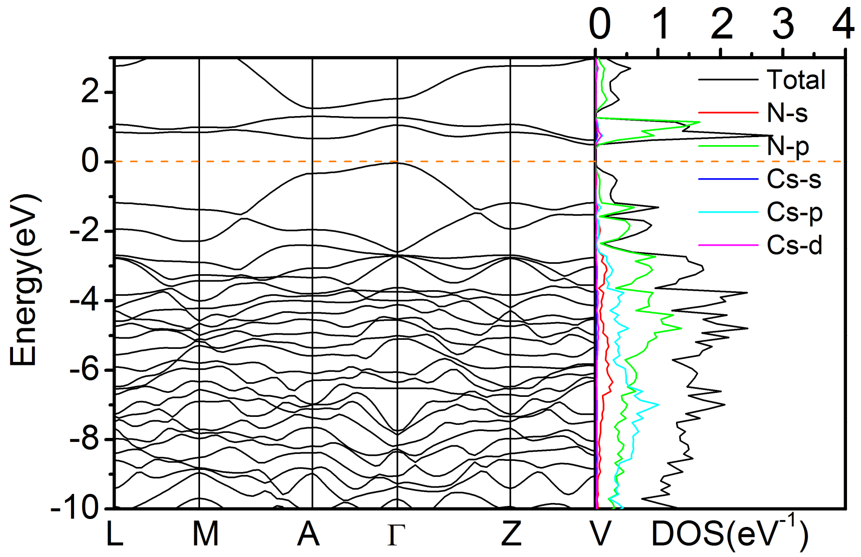

Fig. S14. Electronic band structure (left panel) and projected density of states (PDOS, right panel) of CsN_3 with $C2/m$ symmetry at 100 GPa. The dashed line indicates the Fermi energy.

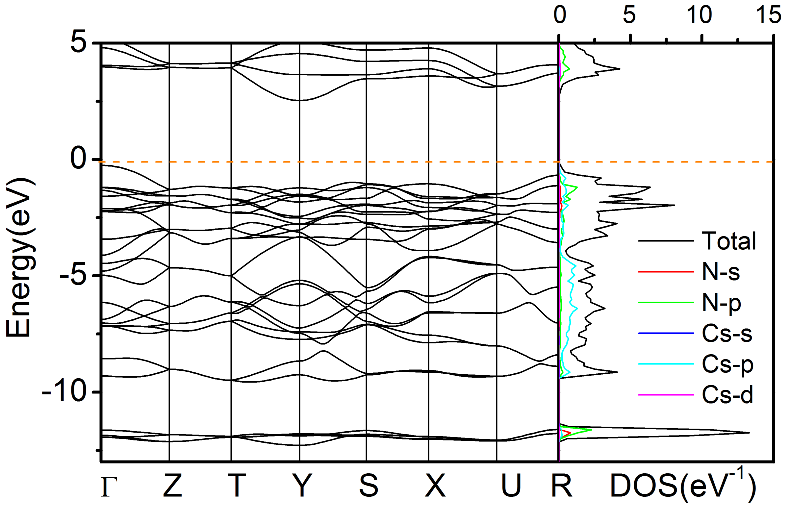

Fig. S15. Electronic band structure (left panel) and projected density of states (PDOS, right panel) of CsN_5 with *Cmc*2_1 symmetry at 50 GPa. The dashed line indicates the Fermi energy.

Supplementary Table

Table S1. Calculated structural parameters of various Cs-N compounds.

	Space group	Lattice Parameters (Å, °)	Atoms	Atomic coordinates (fractional) X	Y	Z
**Cs_3N**	*Cmcm*	*a* = 3.3911	Cs(8f)	0.00000	-0.13871	1.43349
**(50 GPa)**		*b* = 10.4641	Cs(4c)	0.00000	-0.57608	0.75000
		c = 8.4242	N(4c)	0.00000	-0.26047	0.75000
		α=*β*=*γ*=90.0000°				
**Cs_2N**	*C*2/*m*	*a* = 10.8502	Cs(4i)	0.26018	0.50000	0.34453
**(50 GPa)**		b =3.3807	Cs(4i)	0.91680	0.00000	0.10727
		c = 8.2598	N(4i)	0.02903	0.50000	0.60525
		α=β=90.0000°				
		γ = 136.9505°				
**CsN**	*C*2/*m*	*a*= 10.6125	Cs(4i)	0.35173	0.50000	0.80928
**(20 GPa)**		*b*= 3.3835	N(4i)	0.04403	0.50000	0.62581
		c = 4.1495				
		α=*β*=90.0000°				
		γ = 102.9287°				
**CsN**	*P*-1	*a* =4.9212	Cs(2i)	0.97935	0.76213	0.28208
**(50 GPa)**		*b* = 4.9386	Cs(2i)	0.38474	0.30542	0.30212
		c = 5.3148	N(2i)	0.21542	0.75305	0.87954
		α=74.9090°	N(2i)	0.42382	0.88039	0.05554
		β=109.3728°				
		γ= 101.0377°				
**CsN_2**	*C*2/*m*	*a* = 7.9105	Cs(4i)	0.71239	0.00000	0.96850
**(20 GPa)**		*b* = 6.7235	N(8g)	0.44999	-0.30627	0.60216
		c = 3.5422				
		α=*γ*= 90.0000°				
		β= 101.5493°				
**CsN_2**	*C*2/*c*	*a* = 11.07710	Cs(8f)	0.34184	-0.33396	0.21260
**(0 GPa)**		*b* = 6.7982	N(8f)	0.45355	0.06237	0.02036
		c = 5.9044	N(8f)	0.45185	-0.20859	0.68566
		α=*γ*= 90.0000°				
		β= 77.8797°				
**CsN_3**	*I*4/*mcm*	*a* = *b* = 6.6930	Cs(4a)	0.50000	0.50000	0.25000
**(0 GPa)**		*c* = 8.2040	N(8h)	0.87427	0.37427	0.50000
		α=*β*=*γ*= 90.0000°	N(4d)	0.00000	0.50000	0. 50000
**CsN_3**	*C*2/*m*	*a* = 11.3067	Cs(4i)	0.38277	0.00000	0.73364

**(10 GPa)**		*b* =5.4036	Cs(4i)	0.77724	0.00000	0.65536
		c = 4.9467	N(8j)	0.14666	0.87834	0.90961
		α=*γ*= 90.0000°	N(8j)	0.49380	0.61702	0.71962
		β=73.2020°	N(8j)	0.42705	0.25219	0.20005
**CsN_3**	*P*2$_1$/*m*	*a* = 3.5020	Cs(2e)	0.05071	0.25000	0.19007
**(20 GPa)**		*b* =4.9299	N(2e)	0.54949	0.25000	0.67681
		c = 6.6482	N(2e)	0.58104	0.75000	0.17041
		α=*γ*= 90.0000°	N(2e)	0.29788	0.75000	0.46610
		β=100.2791°				
**CsN_3**	*C*2/*m*	*a* = 11.30670	Cs(4i)	0.38277	0.00000	0.73364
**(100 GPa)**		*b* =5.4036	Cs(4i)	0.77724	0.00000	0.65536
		c = 4.9467	N(8j)	0.14666	0.87834	0.90961
		α=*γ*=90.0000°	N(8j)	0.49380	0.61702	0.71962
		β=73.202°	N(8j)	0.42705	0.25219	0.20005
**CsN_5**	*Cmc*2$_1$	*a* = 10.457099	Cs(4a)	0.00000	-0.87531	0.80441
**(50 GPa)**		*b* = 9.506692	Cs(4a)	0.00000	-0.37929	0.86663
		c = 4.77148	N(8b)	0.13918	-0.85348	0.29583
		α=*β*=*γ*=90.0000°	N(8b)	0.29969	-0.55046	0.65793
			N(8b)	-0.28006	-0.71207	0.95633
			N(8b)	0.16852	-0.34499	0.41872
			N(8b)	-0.18207	-0.55538	0.73533
